# Difficult Cases

**Published:** 1887-03-26

**Authors:** 


### Difficult Cases.

[Inquiries and Cases are always welcome for this Column.]

### Acute Mania.

A. M. E. writes:—"William Gostling, who most worthily and efficiently filled a post of great trust at Rawlings' Hotel, from too arduous work and an unfortunate series of shocks to a sensitive nervous system, became the subject of acute mania, and, while so afflicted, behaved with much violence. The case was brought into court about Christmas, but so strong was the testimony to his merits when in health, that much sympathy was expressed, and he was discharged. He is ordered into country air by his hospital doctor, employ shimself in knitting, and is most handy in all ways; a gardener's son, and fond of it; is never idle; a most kindly, cheerful, useful fellow. Will any doctor or person used to such cases take him and look after him? He must not be overworked, but is worth his board. Something could be paid by a sister in service."

[If any of our readers can help this case we will gladly forward their letters to A. M. E.—Ed.]

